# Supplementary material for: Ephrin Signaling Patterns Sensory Neurons During Tissue Homeostasis in Planarians
Source: bioRxiv. 2026 Aug 12:2026.08.11.744258. Preprint. [Version 1] doi: 10.64898/2026.08.11.744258 (PMC13640687; doi:10.64898/2026.08.11.744258)

bioRxiv preprint doi: https://doi.org/10.64898/2026.08.11.744258; this version posted August 12, 2026. The copyright holder for this preprint (which was not certified by peer review) is the author/funder, who has granted bioRxiv a license to display the preprint in perpetuity. It is made available under a CC-BY 4.0 International license.

Supplemental Figure Legends

Supplemental Figure 1: Analysis of genes expressed in *EphR1*⁺ single-cell RNA-seq clusters. (A) Knockdown of *EphR1* resulted in ectopic expression of *scratch*$^+$ and calm2+. (B) CNS architecture revealed by anti-Synapsin staining. Scale bars = 200 µm. (C) Knockdown of *EphR1* resulted in ectopic expression of *cav-1*$^+$ and *nkx2-4*$^+$ cells. Scale bars = 200 µm. All animals are dorsal side up. Experiments were duplicated, with 5 worms per condition and 1 representative worm imaged per condition.

Supplemental Figure 2. A Bayesian inference phylogeny of Ephrin Receptor proteins. Node support values are shown as percentages next to the relevant nodes. *Bf, Brachiostoma floridae; Ce, Caenorhabditis elegans; Ci, Ciona intestinalis; Dm, Drosophila melanogaster; Efn, Ephydatia fluviatilis; Hs, Homo sapiens; Nv, Nematostella vectensis; Sp, Strongylocentrotus purpuratus; Smed, Schmidtea mediterranea*.

Supplemental Figure 3. A Bayesian phylogenetic tree of ephrin ligand proteins. Node support values are shown as percentages next to the relevant nodes. *Bf, Brachiostoma floridae; Ce, Caenorhabditis elegans; Ci, Ciona intestinalis; Dm, Drosophila melanogaster; Efn, Ephydatia fluviatilis; Hs, Homo sapiens; Nv, Nematostella vectensis; Sp, Strongylocentrotus purpuratus; Smed, Schmidtea mediterranea*.

Supplemental Figure 4. Expression patterns of candidate EphRs and ephrin genes. WISH analysis shows expression patterns of the two Eph receptors (EphRs) and three ephrin ligand genes examined to date. Scale bars = 200 µm.

Supplemental Figure 5. Effects of ephrin signaling gene knockdown on peripheral sensory neuron expression and on intestinal and central nervous system architecture. In situ hybridization for *cofilin* and *chat* was used to mark the intestine and the nervous system, respectively. Knockdown of ephrin signaling genes did not result in

bioRxiv preprint doi: https://doi.org/10.64898/2026.08.11.744258; this version posted August 12, 2026. The copyright holder for this preprint (which was not certified by peer review) is the author/funder, who has granted bioRxiv a license to display the preprint in perpetuity. It is made available under a CC-BY 4.0 International license.

obvious defects in intestinal or CNS morphology. Scale bars = 200 µm. Experiments were duplicated, with 5 worms per condition and 1 representative worm imaged.

bioRxiv preprint doi: https://doi.org/10.64898/2026.08.11.744258; this version posted August 12, 2026. The copyright holder for this preprint (which was not certified by peer review) is the author/funder, who has granted bioRxiv a license to display the preprint in perpetuity. It is made available under a CC-BY 4.0 International license.

SUPPLEMENTAL FIGURE 1

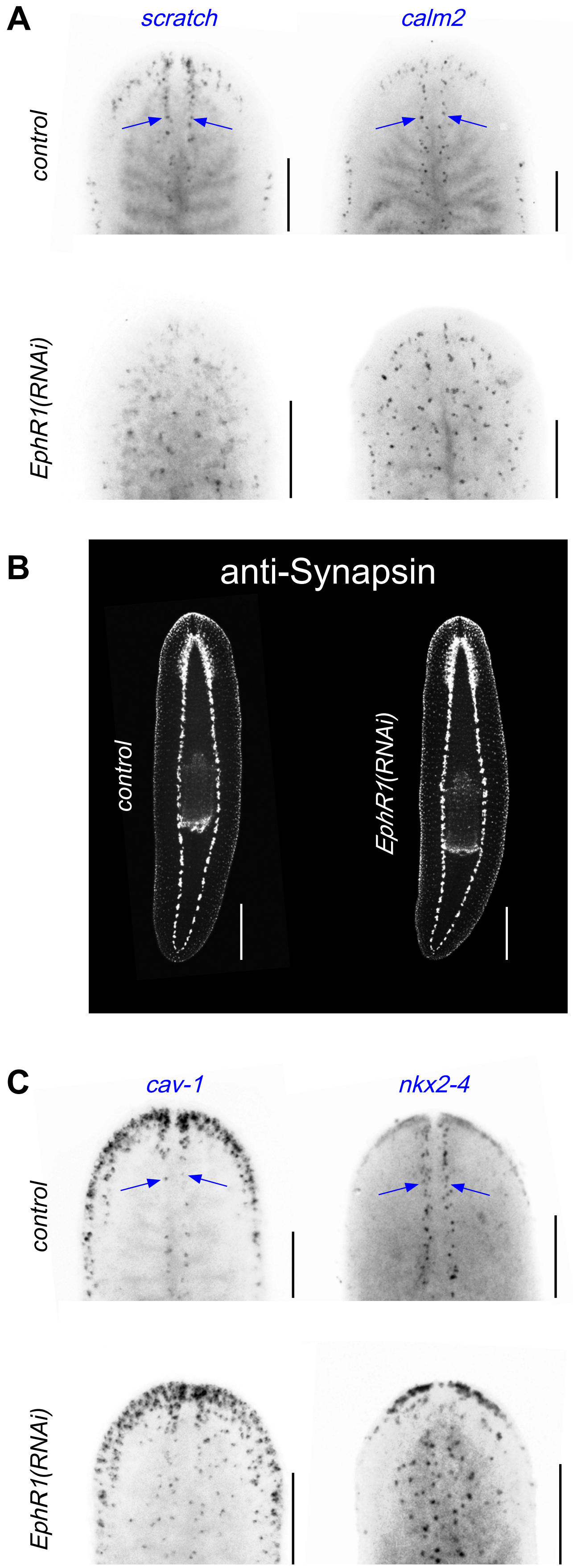

bioRxiv preprint doi: https://doi.org/10.64898/2026.08.11.744258; this version posted August 12, 2026. The copyright holder for this preprint (which was not certified by peer review) is the author/funder, who has granted bioRxiv a license to display the preprint in perpetuity. It is made available under a CC-BY 4.0 International license.

SUPPLEMENTAL FIGURE 2

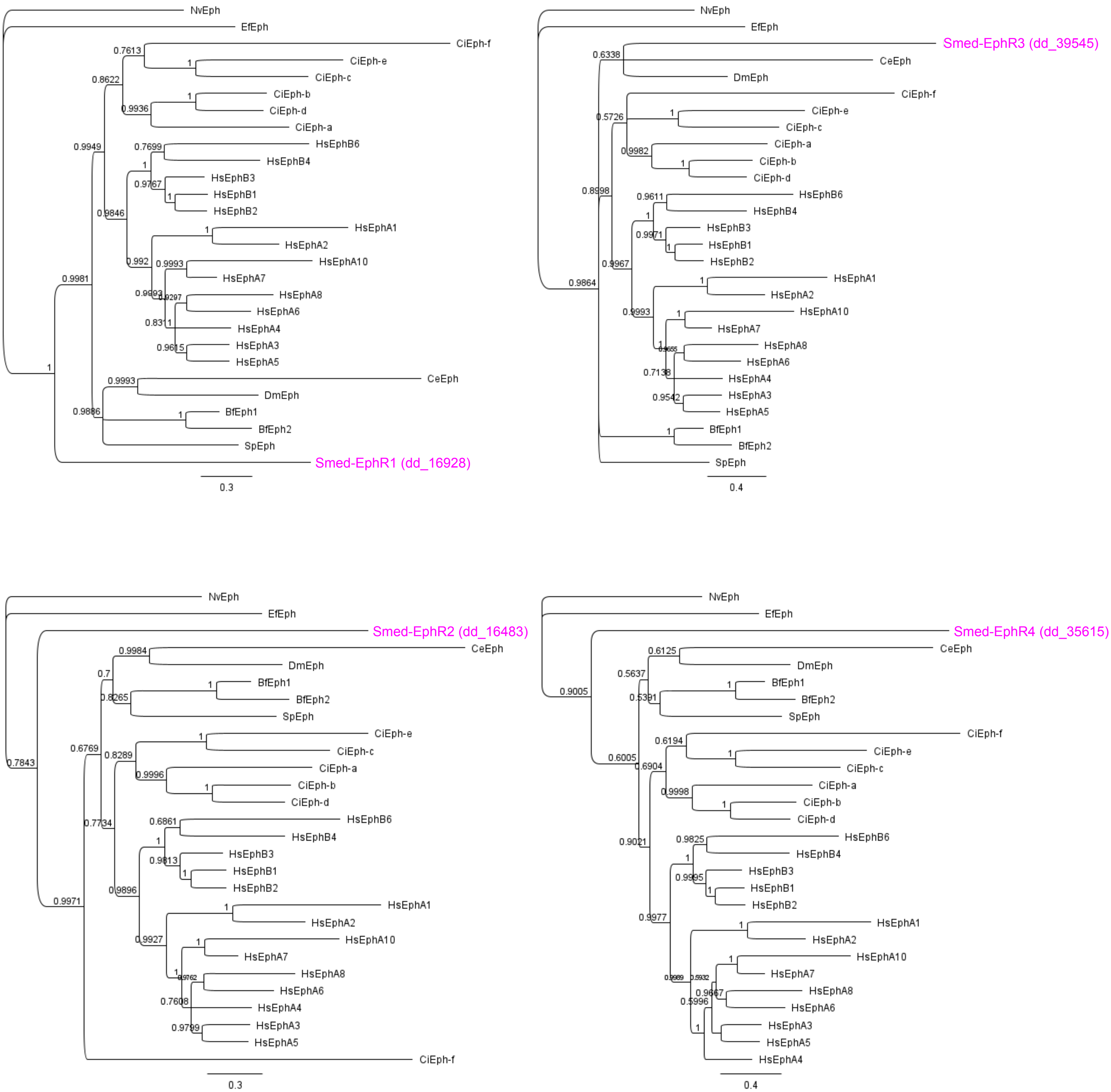

bioRxiv preprint doi: https://doi.org/10.64898/2026.08.11.744258; this version posted August 12, 2026. The copyright holder for this preprint (which was not certified by peer review) is the author/funder, who has granted bioRxiv a license to display the preprint in perpetuity. It is made available under a CC-BY 4.0 International license.

SUPPLEMENTAL FIGURE 3

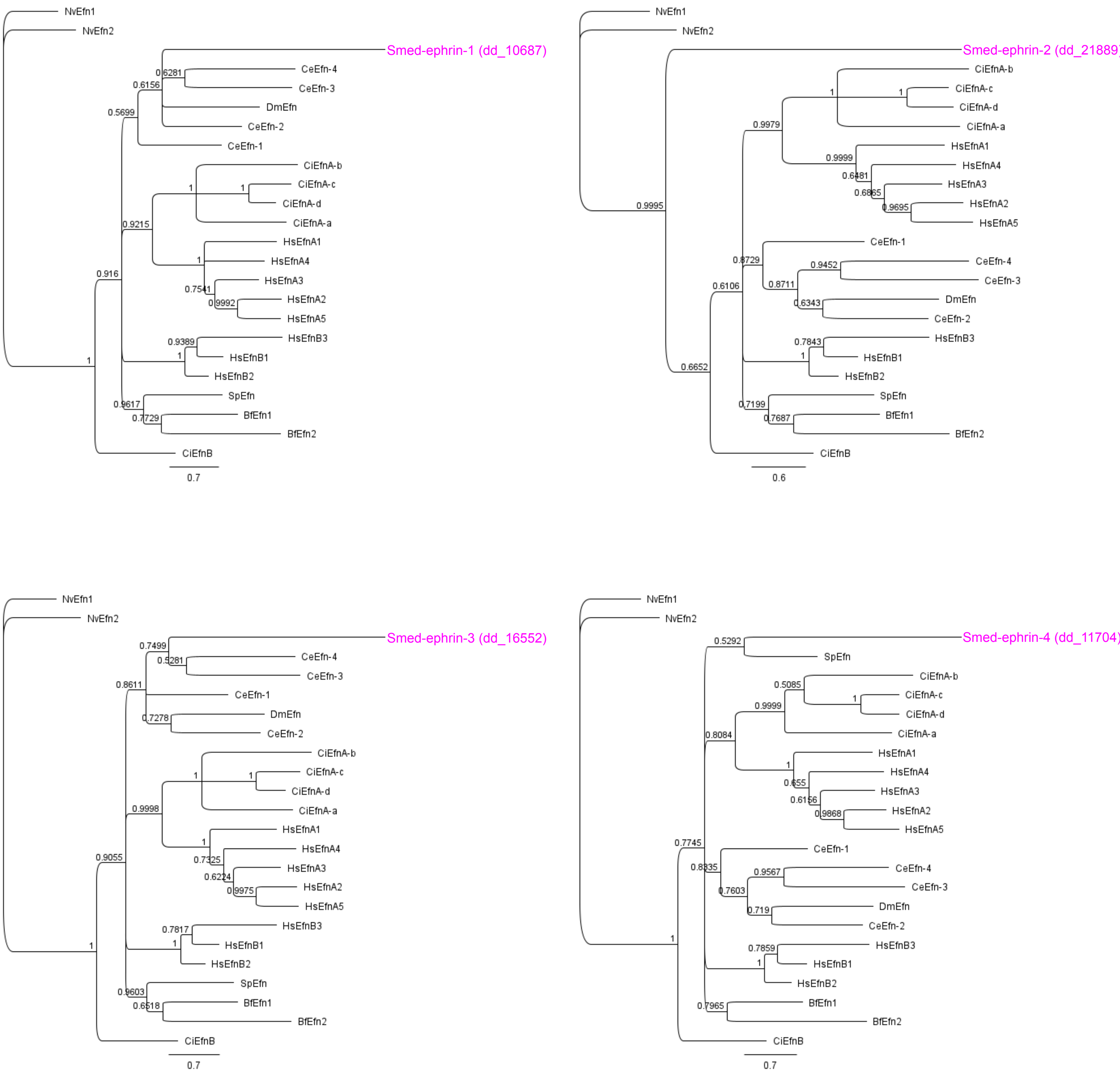

bioRxiv preprint doi: https://doi.org/10.64898/2026.08.11.744258; this version posted August 12, 2026. The copyright holder for this preprint (which was not certified by peer review) is the author/funder, who has granted bioRxiv a license to display the preprint in perpetuity. It is made available under a CC-BY 4.0 International license.

SUPPLEMENTAL FIGURE 4

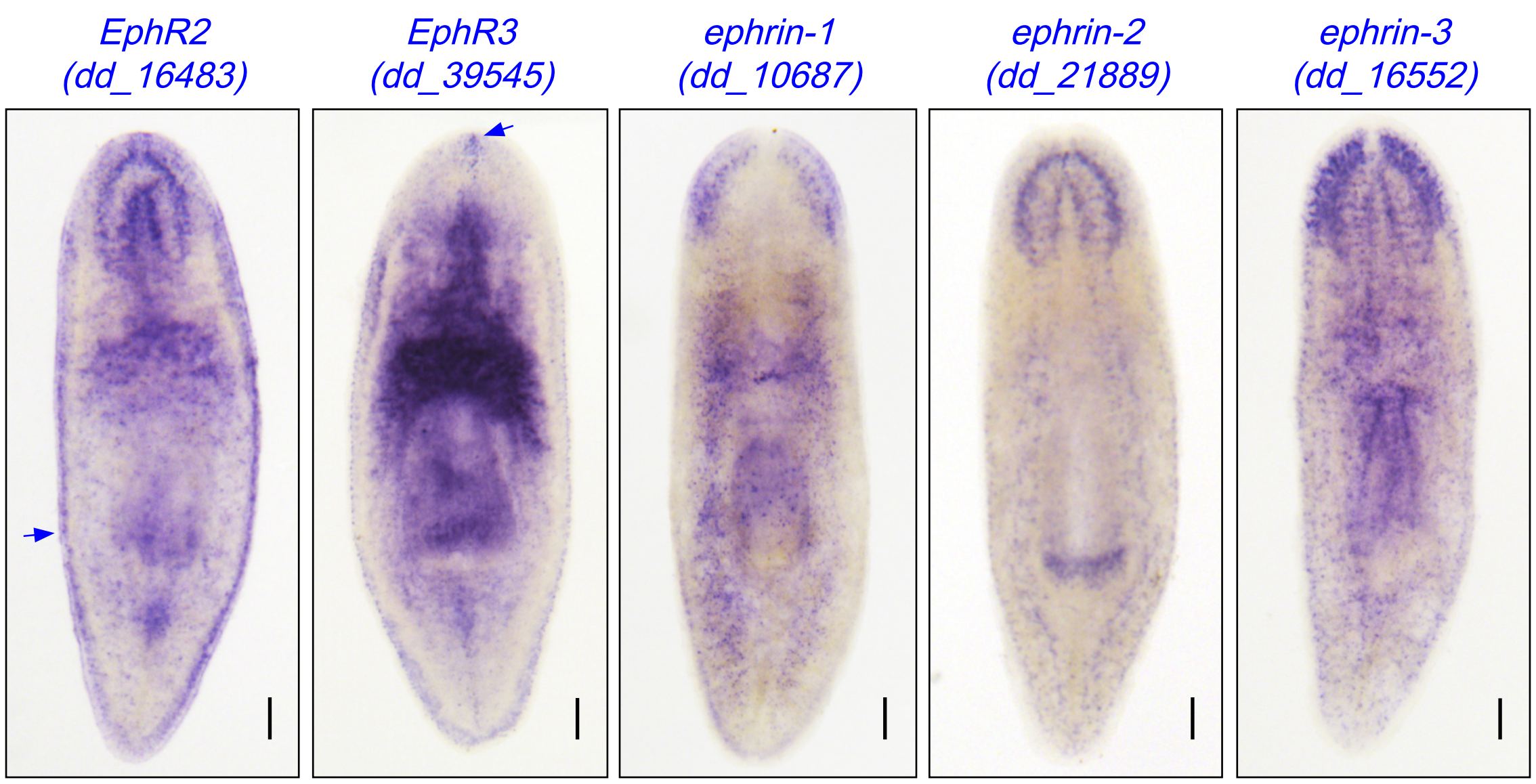

bioRxiv preprint doi: https://doi.org/10.64898/2026.08.11.744258; this version posted August 12, 2026. The copyright holder for this preprint (which was not certified by peer review) is the author/funder, who has granted bioRxiv a license to display the preprint in perpetuity. It is made available under a CC-BY 4.0 International license.

SUPPLEMENTAL FIGURE 5

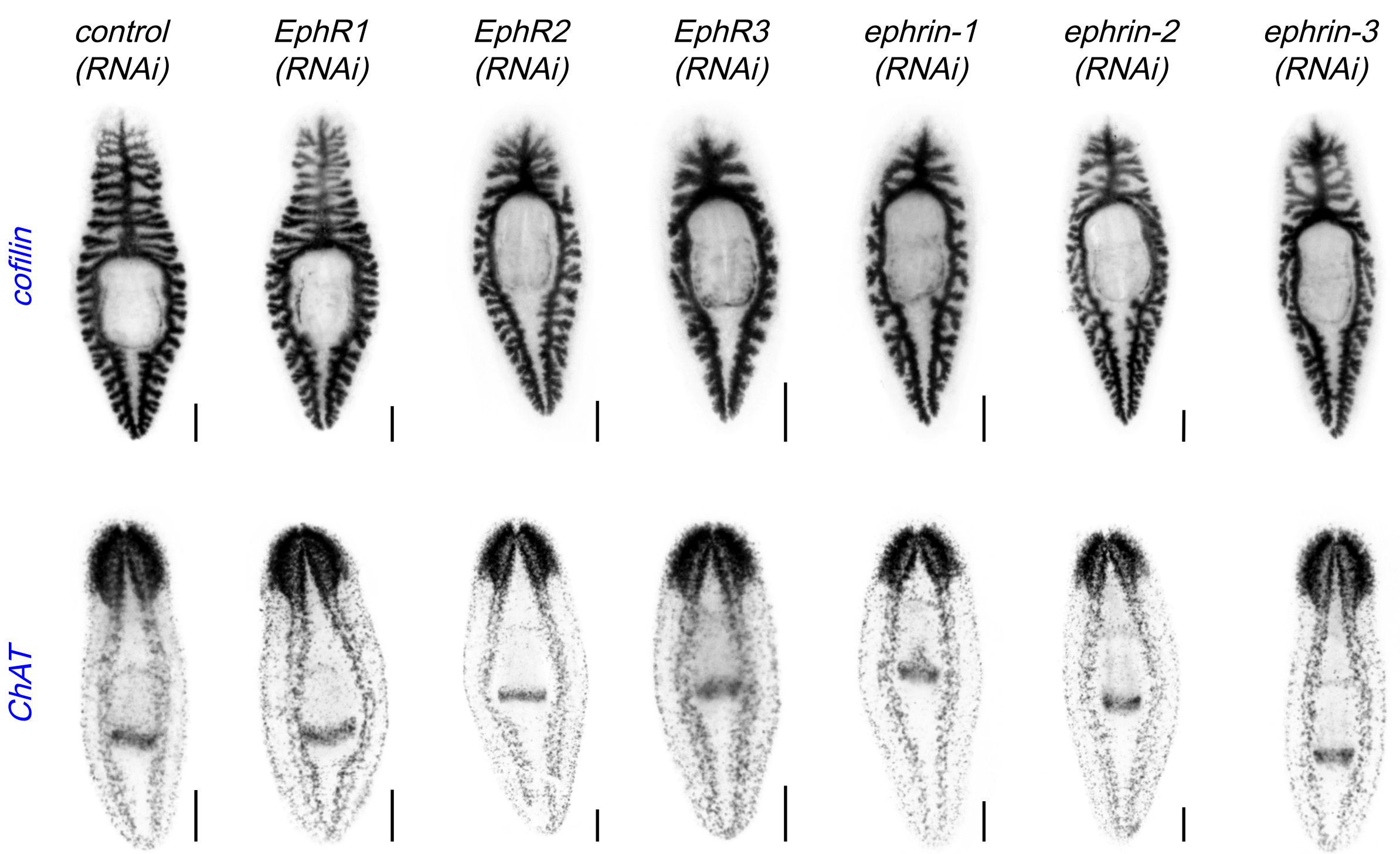

Supplement: Supplementary file 1 [file NIHPP2026.08.11.744258v1-supplement-14.pdf]
